# Automatic Acoustic Target Detecting and Tracking on the Azimuth Recording Diagram with Image Processing Methods

**DOI:** 10.3390/s19245391

**Published:** 2019-12-06

**Authors:** Fan Yin, Chao Li, Haibin Wang, Fan Yang

**Affiliations:** 1 State Key Laboratory of Acoustics, Institute of Acoustics, Beijing 100190, China; yinfan0120@foxmail.com (F.Y.); whb@mail.ioa.ac.cn (H.W.); 2 School of Electronic, Electrical and Communication Engineering, University of Chinese Academy of Sciences, Beijing 100190, China; 3 Laboratory of ImViA, University of Burgundy-France-Comté, 21078 Dijon, France; fanyang@u-bourgogne.fr

**Keywords:** target detecting, target tracking, image processing, principal component analysis, direction of arrival, passive detection, template matching

## Abstract

Passive acoustic target detection has been a hot research topic for a few decades. Azimuth recording diagram is one of the most promising techniques to estimate the arrival direction of the interested signal by visualizing the sound wave information. However, this method is challenged by the random ambient noise, resulting in low reliability and short effective distance. This paper presents a real-time postprocessing framework for passive acoustic target detection modalities by using a sonar array, in which image processing methods are used to automate the target detecting and tracking on the azimuth recording diagram. The simulation results demonstrate that the proposed approach can provide a higher reliability compared with the conventional ones, and is suitable for the constraints of real-time tracking.

## 1. Introduction

**Figure 1:**
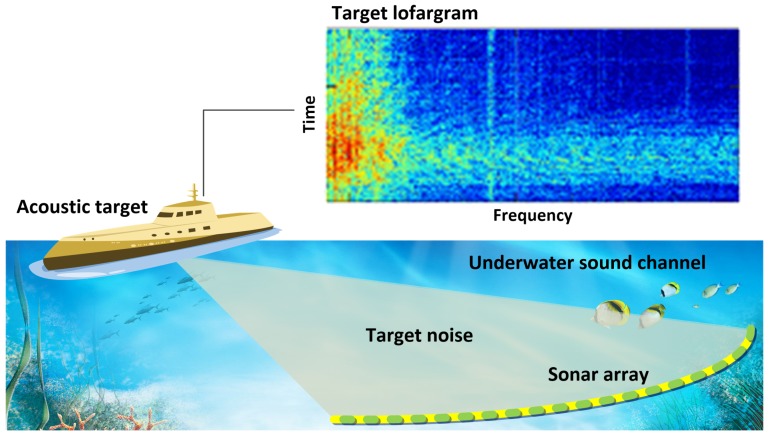
Passive array detection.

The passive sonar detection technique is important for ocean exploration. As shown in Figure 1, the radiated noise of underwater acoustic targets, such as engine noise, active detection pulse, and underwater acoustic communication pulse, can be used by a passive sonar array for direction estimation and tracking.

Direction of arrival (DOA) estimation is a crucial topic in the field of passive sonar information processing [1,2,3]. Its primary mission is to estimate the performance parameters of space target signal (e.g., the number of target signal sources, direction of arrival, frequency and polarization of signal sources, etc.) by using the received array data. A complete DOA estimation system mainly includes three aspects:

(a) Target space: this space is mainly composed of the target incident signal source and the actual environment. The DOA estimation systems capture the underwater acoustic information via some sensors, e.g., optic, pressure, or vector hydrophones.
(b) Observation space: mainly refers to the use of arrays placed in space according to certain rules (e.g., uniform linear or spherical arrays) in advance to obtain the array information of the target incident signal source.
(c) Estimation space: the parameter information of the target signal source obtained via the sonar array is extracted by the DOA estimation algorithm.

The conventional beamforming (CBF) method is recognized as one of the earliest and classical DOA estimation algorithms for sensor arrays, which is also commonly known as the Bartlett beamforming method [4,5,6,7]. It is a simple extension of space domain in the traditional time domain Fourier spectrum estimation technique. Its aim is to replace the information data obtained from each sensor element in the space domain with the time domain information. Unfortunately, like Fourier constraints in time domain, the resolution of CBF methods is constrained by the physical aperture of arrays, often referred to as the Rayleigh limit, i.e., in the case of multiple space target signal sources within the same beam width, it is quite difficult to achieve high resolution. For the purpose of high accuracy, based on the conversion relationship between time domain processing and spatial processing of target signals, many nonlinear spectral estimation techniques have been extended to spatial spectral estimation techniques, and high resolution spectral estimation methods have been developed, e.g., Maximum Entropy Method [8], Minimum Variance Method [9], and harmonic analysis method [10]. However, most of them are not suitable for practical applications, especially for passive detection modalities, because the resolution is improved only when the form and parameters of the received signals are known.

**Figure 2:**
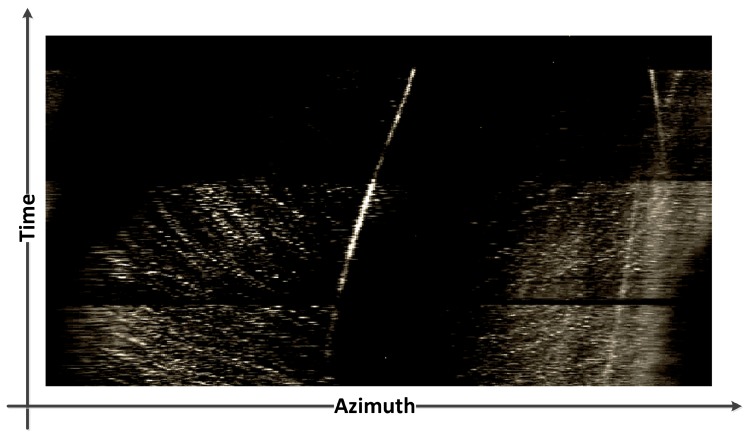
An example of acoustic target azimuth recording diagram.

Based on the CBF-like techniques, azimuth recording diagram is developed to facilitate DOA estimation by visualizing the received signal information. As shown in Figure 2, the azimuth recording diagram is a spatial coordinate system whose x- and y-axis correspond to the azimuth and time, respectively. At each node, the gray levels are used to represent the power of the beamforming. If there exists an underwater acoustic source in some direction, the output power of the beamforming in that direction will be higher than that in the other directions. If the noise source persists, a stable and bright trajectory will appear on the diagram, and the trajectory varies when the target sound source moves. In realistic applications, if a piece of bright trajectory is detected on the azimuth recording diagram, the corresponding azimuth can be considered as having a target, and then we can start to track it along the trajectory manually or automatically.

Due to its benefits of stability and convenience, this technique is widely used. However, detecting the target on the diagram by using naked eyes is far from easy, because the received signal of a sonar array is distorted by the ambient noise, which results in a large number of random image noises, especially in a low SNR (Signal-to-Noise Ratio) environment [11,12,13]. Additionally, with the noise in the image level, there is a certain probability that the ambient noise peak is misjudged as the target noise, resulting in tracking failure. A postprocessing cycle is therefore necessary to mitigate the ambient noise in the image level or enhance the interested image patterns.

Our work focuses on the explorations of robust acoustic target localization methods. This paper is an extension of our previous work presented in the ICSIP 2019 [14]. It presents an image postprocessing framework for automatic target detecting and tracking within the underwater acoustic azimuth recording diagram, where advanced image processing techniques are innovationally applied for azimuth recoding diagram analysis. The proposed framework consists of three cycles: target detecting, pattern enhancement and automatic azimuth tracking. The highlights of our work include the following.

(a) The first step of the postprocessing framework is to find the weak trajectory inundated with the noise on the diagram quickly and accurately. This paper realizes the automatic trajectory detecting via template matching. To this end, we propose a feasible trajectory template generation method allowing users to customize the template set for different requirements.
(b) Pattern enhancement is the second step. Conventional target tracking methods based on the azimuth recording diagram use the local power optima of the beams performed with different arrival directions as the patterns to track, which is easy to deviate from the trajectory due to the influences of ambient noise, so we enhance the trajectory patterns by using spatial separation methods, which significantly facilitates the tracking tasks.
(c) Finally, the pattern enhancement method presented in this paper may lead to the azimuth migration if the target direction varies fast. An azimuth correction strategy is therefore conceived to improve the accuracy of the DOA estimation.

The experiment of this work is conducted by the simulated data. The proposed method is evaluated by comparing with the conventional detecting and tracking technique. The simulation results demonstrate that the proposed approach has a high stability and reliability by suppressing noise when tracking target with a strong Gaussian white noise, and its efficiency meets the real-time data processing requirements.

The reminder of this paper is organized as follows. Section 2 reviews the related works of azimuth recording diagrams. Section 3 and Section 4 present, respectively, the proposed automatic detection and tracking methods for the azimuth recording diagrams. Section 5 evaluates the new approaches with simulation data. Finally, some discussions and conclusions are made in Section 6.

## 2. Related Work

The underwater signal propagation can be modeled as

(1) $$r\left(t\right)=s_{o}\left(t\right)+n\left(t\right)=h\left(t,\tau \right)*s_{i}\left(t\right)+n\left(t\right)$$

where *t* is time, $s_{i}$ is the baseband signal waveform, and *n* is the additive noise. h(t,τ) is the time-varying multipath channel impulse response. Berger, C. R. et al. [15] defined h(t,τ) as

(2) $$h\left(t,\tau \right)=\sum_{j=1}^{L}A_{j}\left(t\right)\delta \left(\tau -\tau_{j}\left(t\right)\right)$$

Equation (2) is a simplified description of the classical Wide-Sense Stationary Uncorrelated Scattering (WSSUS) model [16], and it approximates the underwater acoustic channel by using *L* dominant discrete paths. $A_{j}$ refers to the path amplitudes, which changes with the delays as the attenuation is related to the distance traveled as well as the physics of the scattering and propagation processes. With Equation (2), we can simplify Equation (1) to

(3) $$r\left(t\right)=\sum_{j=1}^{L}A_{j}\left(t\right)s_{i}\left(t-\tau_{j}\left(t\right)\right)+n\left(t\right)$$

Equation (3) shows that the underwater acoustic signals may be distorted by (a) the multipath effect caused by the sea-surface and bottom reflections; (b) propagation attenuations; and (c) ambient noise such as random noise, flow noise, radiated noise of the third-party artificial equipment, and self noise. Furthermore, the relative motions between the signal source and receiving sensors lead to the Doppler effect, further complicating the mechanisms of the signal distortions [17,18,19]. Therefore, retrieving the desired information directly from the distorted signals is difficult.

Passive acoustic detections are used to estimate the directions by analyzing the power of the synthetic beams from different directions. The conventional (or Bartlett) beamformer is a natural extension of the classical Fourier-based spectral analysis [20] to sonar array data and has been widely used. For arbitrary geometric arrays, the algorithm maximizes the beamforming output power of a given input signal. Given the direction of arrival θ, the received array data with additive noise can be written as

(4) $$x\left(t\right)=a\left(\theta \right)r\left(t\right)=a\left(\theta \right)\sum_{j=1}^{L}A_{j}\left(t\right)s_{i}\left(t-\tau_{j}\left(t\right)\right)+a\left(\theta \right)n\left(t\right)$$

where x(t) can be considered as a multichannel random process, $a\left(\theta \right)={\left[a_{1}\left(\theta \right),\cdots ,a_{N}\left(\theta \right)\right]}^{T}$ is the steering vector, and *N* is the number of elements in a geometric array. Therefore, the problem of maximizing the output power can be expressed as

(5) $$\begin{array}{cc} max_{W}E\left\{W^{T}x\left(t\right)x^{T}\left(t\right)W\right\} & =max_{W}W^{T}E\left\{x\left(t\right)x^{T}\left(t\right)\right\}W \end{array}$$

(6) $$\begin{array}{c} =max_{W}{\left\{E|s\left(t\right)\right|}^{2}|W^{T}{a\left(\theta \right)|}^{2}+\sigma^{2}{\left|W\right|}^{2}\} \end{array}$$

where the assumption of spatially white noise is used. *W* is the weight vector, and different beamforming approaches correspond to different choices of *W*; E{} denotes the operation of the statistical expectation; $E\{x\left(t\right)x^{T}\left(t\right)\}$ is the source covariance matrix; $\sigma^{2}$ is eigenvalue; and the last covariance structure is a reflection of the noise having a common variance $\sigma^{2}$ at all sensors and being uncorrelated among all sensors.

To obtain a non-negative solution, the norm of *W* is constrained to |W|=1. The solution can therefore be expressed as

(7) $$W_{BF}=a\left(\theta \right)/\sqrt{a{\left(\theta \right)}^{T}a\left(\theta \right)}$$

where BF means conventional Beamformer. The above weight vector can be interpreted as a spatial filter, which makes the delays (and possible attenuations) experienced by signals on different sensors equal, thus maximizing the combination of their respective contributions.

**Figure 3:**
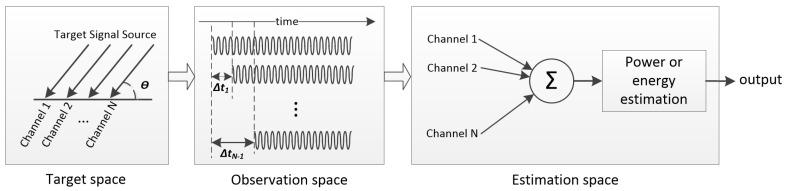
Classical beamforming framework.

Figure 3 displays the framework of classical beamforming. For irregular sensor arrays, the steering vector a(θ) is a function of the azimuth of arrival θ and the array shape. Let the azimuth of arrival θ be the angle between the x-axis and the arrival direction, the *i*-th element of a(θ) will be

(8) $$a_{i}\left(\theta \right)=e^{2\pi f\Delta t}$$

with

(9) $$\Delta t=\frac{\left(x^{\prime }-x_{o}\right)\cos\theta +\left(y^{\prime }-y_{o}\right)\sin\theta }{c}$$

where *f* is the frequency of the baseband signal, $\left(x_{o},y_{o}\right)$ and $\left(x^{\prime },y^{\prime }\right)$ are, respectively, the coordinates of the reference and interested sensors, and *c* is the sound velocity. Now, the array data can be visualized as the azimuth recording diagram by computing the short-time power with Equations (4)–(9). The power values over the azimuths of each short-time data is defined as a snap. We describe programmatically computing process of the azimuth recording diagram as follows,

(a) for all the directions of arrival, compute the steer vectors a(θ) with Equation (8), then the weight vectors $W_{BF}\left(\theta \right)$ using Equation (7);
(b) for all the weight vectors, compute the expectations of the output power with Equation (5); and
(c) perform the azimuth recording diagram by establishing a time-azimuth space coordinate system, in which the image intensity is used to represent the power level of the synthetic signals over time and azimuth.

## 3. Target Detection

The first step of the proposed postprocessing framework is to detect the trajectory automatically. From the view point of image pattern recognition, detecting the target via azimuth recording diagram is a pattern detection task, and the interested patterns are those trajectories performed by the power peaks. We address this problem by using the template matching technique.

### 3.1. Generation Model of Trajectory Templates

The preliminary preparation for template matching is to establish a suitable matching template set. To do this, some parameters of the sonar systems should be provided by the users: (a) the minimum effective distance $d_{min}$ of sensor array, (b) the maximum navigation speed $v_{max}$ of the target to be observed, (c) the system response time delay $T_{min}$ (the maximum time delay for the system to react to the target after it appears), and (d) the azimuth interval step Δθ.

**Figure 4:**
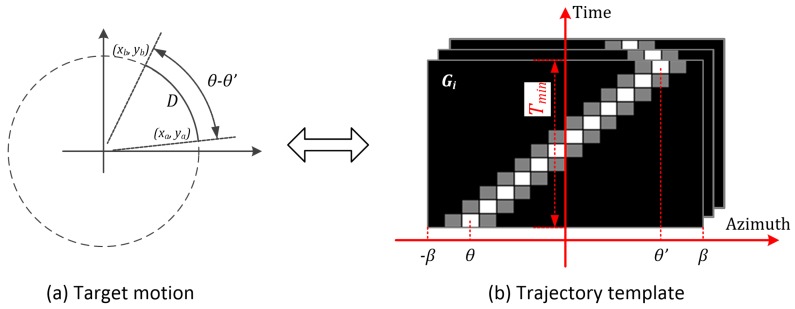
An example of template generation.

Next, initialize the template set *G*, which contains all two-dimensional trajectory templates. The templates $G_{i}$ correspond to the trajectories with different forms. As shown in Figure 4, the x-axis of $G_{i}$ corresponds to the azimuth, $d_{min}$ is the minimum effective distance of the desired sensor array, $T_{min}$ is the minimum system response time delay, *D* is the distance that the target moves from $\left(x_{a},y_{a}\right)$ to $\left(x_{b},y_{b}\right)$, and β is the template $G_{i}$. Let the scale of the x-axis be [−β,β], which represents the maximum azimuth-varying range of the target in the response time of the system. β is computed by

(10) $$\beta =\frac{90\times v_{max}\times T_{min}}{\pi \times d_{min}}$$

The y-aixs of $G_{i}$ corresponds to time, and each discrete time is the arrival time of the sampling snapshot within $T_{min}$. The gray values of $G_{i}$ correspond to the power of the synthetic signals at <ϑ,t>, where ϑ and *t* denote azimuth and time, respectively. Because the acoustic target usually moves slowly, we hypothesize that its azimuth changes linearly over time and describe the corresponding trajectory as

(11) t=Aϑ+B

with

(12) $$\begin{array}{c} A=\frac{T_{min}}{\theta -\theta^{\prime }}\\ B=t^{\prime }-\frac{T_{min}}{\theta -\theta^{\prime }}\times \theta^{\prime } \end{array}$$

*A* and *B* are the parameters of the linear model to be computed. As shown in Figure 4, θ and $\theta^{\prime }$ are the initial and ending azimuths of the simulated trajectory, respectively. $t^{\prime }$ is the time of $\theta^{\prime }$. $T_{min}$ is the maximum system response time delay. The template $G_{i}$ can therefore be discretely modeled as

(13) $$G_{i}\left(\vartheta ,t\right)=\left\{\begin{array}{cc} 1 & \;t=[\frac{\vartheta -\theta^{\prime }}{\theta -\theta^{\prime }}T_{min}+t^{\prime }]\\ \eta & \;t=[\frac{\vartheta -\theta^{\prime }}{\theta -\theta^{\prime }}T_{min}+t^{\prime }]\pm \Delta \theta \\ 0 & \;\mathrm{otherwise} \end{array}\right.$$

where η∈[0,1] is a user-defined constant, and this paper sets it as 0.5. Δθ is the azimuth interval step.

### 3.2. Matching Process

#### 3.2.1. Two-Dimensional Matched Filter

We base the target trajectory detection on the two-dimensional (2-D) matched filter. Suppose that the transfer function and impulse response of the matched filter are H(u,v) and ℏ(ϕ,t), respectively, and the output is written as

(14) $$y\left(\phi ,t\right)=(I\left(\phi ,t\right)+\bar{n}\left(\phi ,t\right))*\hbar \left(\phi ,t\right)$$

where y(ϕ,t) is the output of the filter, I(ϕ,t) is the baseband signal, and $\bar{n}\left(\phi ,t\right)$ is the additive noise in the image level. We, respectively, write the power spectral density of I(ϕ,t) and the image-level noise $\bar{n}\left(\phi ,t\right)$ as

(15) $$F\left(u,v\right)=\int_{-\infty }^{\infty }\int_{-\infty }^{\infty }I\left(\phi ,t\right)e^{-j(u\phi +vt)}d\phi dt$$

and

(16) $$N\left(u,v\right)=\int_{-\infty }^{\infty }\int_{-\infty }^{\infty }\bar{n}\left(\phi ,t\right)e^{-j(u\phi +vt)}d\phi dt$$

where *u* and *v* correspond to the frequencies of the diagram within the different dimensions. With inverse Fourier transformation, the instantaneous output of the filter at <φ,τ> is rewritten as

(17) $$y\left(\phi ,t\right)=\int_{-\infty }^{\infty }\int_{-\infty }^{\infty }H\left(u,v\right)\left(F\left(u,v\right)+N\left(u,v\right)\right)e^{j(u\varphi +v\tau )}dudv$$

Now we have the instantaneous output signal-to-noise ratio (SNR) of the filter as

(18) $$SNR\left(\varphi ,\tau \right)=\frac{{\left[\int_{-\infty }^{\infty }\int_{-\infty }^{\infty }H\left(u,v\right)F\left(u,v\right)e^{j(u\varphi +v\tau )}dudv\right]}^{2}}{{\left[\int_{-\infty }^{\infty }\int_{-\infty }^{\infty }H\left(u,v\right)N\left(u,v\right)e^{j(u\varphi +v\tau )}dudv\right]}^{2}}$$

Suppose $\bar{n}\left(\phi ,t\right)$ is solely a Gaussian white noise of power density $N_{o}/2$, Equation (18) can be simplified to

(19) $$SNR\left(\varphi ,\tau \right)=\frac{2{\left[\int_{-\infty }^{\infty }\int_{-\infty }^{\infty }H\left(u,v\right)F\left(u,v\right)e^{j(u\varphi +v\tau )}dudv\right]}^{2}}{N_{o}\int_{-\infty }^{\infty }\int_{-\infty }^{\infty }{\left|H\left(u,v\right)\right|}^{2}dudv}$$

The optimal matched filter can be obtained by maximizing Equation (19). According to Cauchy–Bunyakovsky–Schwarz inequality, we have

(20) $${\left[\int_{-\infty }^{\infty }\int_{-\infty }^{\infty }H\left(u,v\right)F\left(u,v\right)e^{j(u\varphi +v\tau )}dudv\right]}^{2}\leq \int_{-\infty }^{\infty }\int_{-\infty }^{\infty }{\left|H\left(u,v\right)\right|}^{2}dudv\int_{-\infty }^{\infty }\int_{-\infty }^{\infty }{\left|F\left(u,v\right)\right|}^{2}dudv$$

therefore, the optimal output SNR is

(21) $$SNR_{opt}=\frac{2}{N_{o}}\int_{-\infty }^{\infty }\int_{-\infty }^{\infty }{\left|F\left(u,v\right)\right|}^{2}dudv$$

the optimal SNR is achieved when

(22) $$H\left(u,v\right)=k{\left[F\left(u,v\right)e^{j(u\varphi +v\tau )}\right]}^{*}=kF^{*}\left(u,v\right)e^{-j(u\varphi +v\tau )}$$

The 2-D matched filter of Equation (22) represents the only type of linear 2-D filter, which maximizes the output SNR.

#### 3.2.2. Implementation of the Matching Process

**Figure 5:**
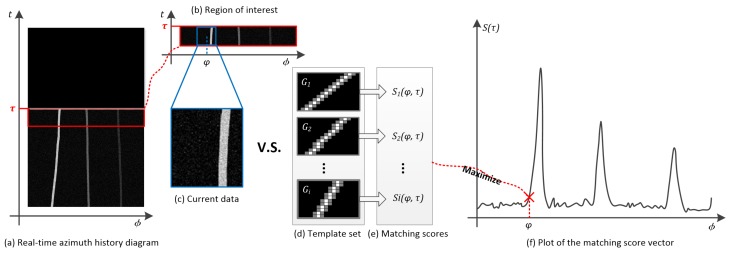
Implementation of matching process.

The azimuth recording diagram is updated snap by snap in real-time applications. As shown in Figure 5a, we first extract the ROI (Region of Interest) *M* from the screen of the diagram. *M* is essentially a matrix that has the same row size with the template $G_{i}$ mentioned in Section 3.1. The columns of *M* correspond to the angle ranging from −π to π, for example.

Next, *M* is spatially filtered. According to the optimal filters presented in Section 3.2.1, we assign directly the generated templates $G_{i}$ to the impulse response of the filters for different trajectory templates. Therefore, the output of the filter can be discretely expressed as

(23) $$S\left(\phi ,t\right)=\sum_{p=\phi -\beta }^{\phi +\beta }\sum_{q=t-T_{min}}^{t}G_{i}\left(p-\phi ,t-q\right)I_{n}\left(p,q\right)$$

As shown in Figure 5c–e, the matching results of all the templates $G_{i}$ perform a matching score vector $S\left(\phi ,t\right)=<S_{1}\left(\phi ,t\right),S_{2}\left(\phi ,t\right),\ldots ,S_{i}\left(\phi ,t\right)>$ over azimuths ϕ. The maximum of S(ϕ,t) is selected as the instantaneous output of the matching process at (ϕ,t).

Figure 5f depicts an example of matching scores S(t) over the azimuths ϕ. The targets are detected with matching score peaks. To do this, S(t) is first smoothed by using a mean filter:

(24) $$\bar{S}\left(\phi ,t\right)=\frac{1}{N}\sum_{k=-\frac{N-1}{2}}^{\frac{N-1}{2}}S\left(\phi +k\Delta \phi ,t\right)$$

where Δϕ=Δθ refers to the azimuth interval step. Next, a target exists when a peak of $\bar{S}\left(t\right)$ is higher than a user-defined threshold ϵ. This determination condition is described as

(25) $$D\left(\phi ,t\right)\in \{\bar{S}\left(\phi ,t\right)|\bar{S}\left(\phi -\Delta \phi ,t\right)\leq \bar{S}\left(\phi ,t\right)\leq \bar{S}\left(\phi +\Delta \phi ,t\right)\;\mathrm{and}\;\bar{S}\left(\phi ,t\right)\geq \epsilon \}$$

Equation (25) includes two constraints: (1) $\bar{S}\left(\phi -\Delta \phi ,t\right)\leq \bar{S}\left(\phi ,t\right)\leq \bar{S}\left(\phi +\Delta \phi ,t\right)$, which defines the peaks (the value of $\bar{S}\left(\phi ,t\right)$ is higher than either of the neighbors), and (2) $\bar{S}\left(\phi ,t\right)\geq \epsilon$, which is used to judge whether the peaks are higher than the user-defined threshold. Once both the constraints are satisfied, we have the azimuth of the target $\hat{\phi }\left(t\right)$ as

(26) $$\hat{\phi }\left(t\right)=\phi_{D}+\theta^{\prime }$$

where $\phi_{D}$ is the azimuth satisfying the constraint in Equation (25) and $\theta^{\prime }$ is the ending azimuth of the simulated trajectory of the optimal template at $\left(\phi_{D},t\right)$.

## 4. Target Enhancement and Tracking

The underwater acoustic target azimuth recording diagram is essentially a fusion of useful information and interference noise, so it can be projected into useful information and interference noise subspaces. This paper enhances the azimuth recording diagram using principal component analysis (PCA), which has been used in the sonar or radar systems [21,22,23,24,25].

**Figure 6:**
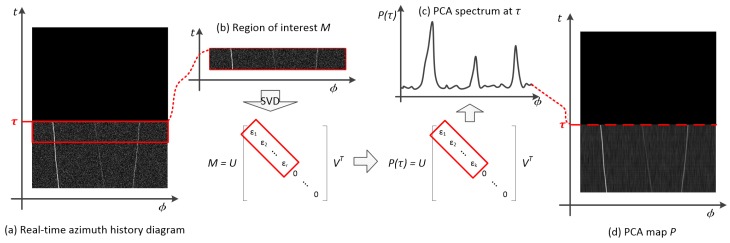
Principal component analysis of azimuth history diagram.

As shown in Figure 6a,b, when a new snap comes, the current region of interest M(τ) is first decomposed into the form of $M\left(t\right)=U\left(t\right)\varepsilon \left(t\right)V{\left(t\right)}^{T}$ via singular value decomposition (SVD), where U(t) and V(t) are the left and right singular value vectors of M(t), respectively, and ε is the singular value matrix of M(t). This paper makes the singular value decompositions by using Jacobi’s method [26,27]. The diagonal elements of ε(t) are singular values of M(t) arranged in descending order, and the other elements are 0. Next, the first *k* singular values are kept to reconstruct M(t):

(27) $$P\left(t\right)=U\left(t\right)\varepsilon^{\prime }\left(t\right)V^{T}\left(t\right)$$

Equation (27) projects *M* into signal subspace, and the projected matrix P(t) is the PCA spectrum of the snap at *t*. The principal component map *P* of the diagram shown in Figure 6d is obtained by repeating this process snap by snap. In this time-azimuth coordinate system, the image intensity is used to represent the spectral amplitude of the principal component spectral.

Now, we can start to track the target trajectories. The acoustic target is used to being automatically tracked in the azimuth recording diagrams by searching for the maximum power of the received signal over the azimuths, then the corresponding azimuth is considered as the current target azimuth. Despite high stability, the peaks of PCA spectra cannot be considered as the real azimuth of the sound source, because enhancing the azimuth recording diagrams with PCA is essentially to alter the optimizing function from maximizing the power into maximizing the power dependency within a certain period, resulting in the tracking migrations. More precisely, the singular value vectors *U* and *V* of *M* are computed by decomposing $MM^{T}$ and $M^{T}M$ via eigen decomposition, respectively. That is, PCA analyzes the relationship among the power values within the horizontal and vertical directions instead of along the trajectories. Therefore, when there exists some angle difference between the axis and trajectory, an azimuth tracking misplacement occurs.

**Figure 7:**
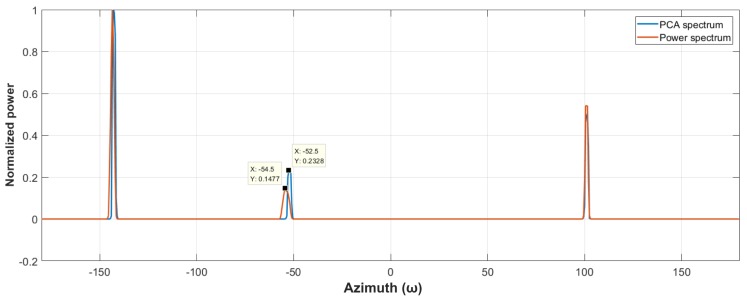
Power and principal component analysis (PCA) spectra of the acoustic target without ambient noise.

Figure 7 compares the power and PCA spectra of the same simulated target, in which there is no ambient noise. We can see that an azimuth misplacement exists between them, mainly at −50°, so if a high-accuracy positioning result is desired, it is not enough to trace the PCA spectrum only. Therefore, when a snap comes, we first determine the position of the maximum peak value in the PCA spectrum in order to ensure the tracing stability. Next, the power spectrum is smoothed with the meaning filter. Finally, the optima of the smoothed power spectrum around the maximum peak of the PCA spectrum is computed within a user-defined range. In this way, the search criteria return back to the optimal power again, therefore the target can be tracked stably and a more precise azimuth can be obtained as well.

## 5. Experiments

### 5.1. Target Detection with Template Matching

**Figure 8:**
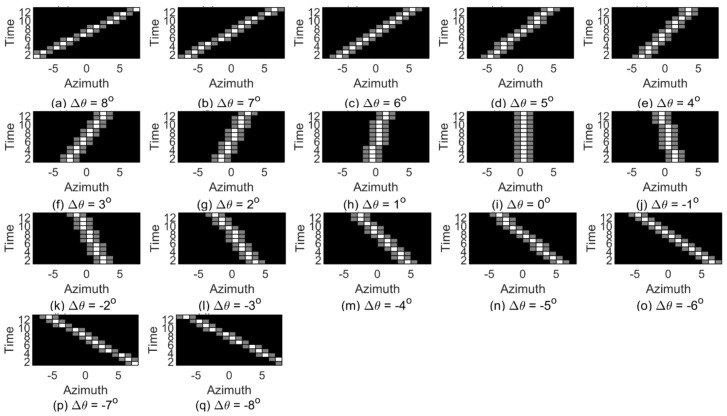
Target azimuth trajectory matching template.

First, a template set of target azimuth trajectory is established by using the method proposed in Section 3. As shown in Figure 8, the set *G* contains 17 2-D matching templates, and each one corresponds to a change state of target azimuth in the system response time $T_{min}=13$ s. It is supposed that the maximum speed of the targets is 40 kn (~74 km per hour) and the minimum effective distance of the system is 2 km. The azimuth range of $G_{i}$ can be approximately computed as $\beta =\left(90\times v_{max}\times T_{min}\right)/\left(\pi \times d_{min}\right)\approx 3.83^{\circ }$. This paper sets β as 4° for the convenience of calculating. For any $G_{i}$ (i=1,2,3,…,17), set the initial azimuth state of the target $\theta =\left(i-9\right)\times \Delta \theta_{step}$ and the end azimuth $\theta^{\prime }=\left(9-i\right)\times \Delta \theta_{step}$, then it can be computed via Equation (13).

**Figure 9:**
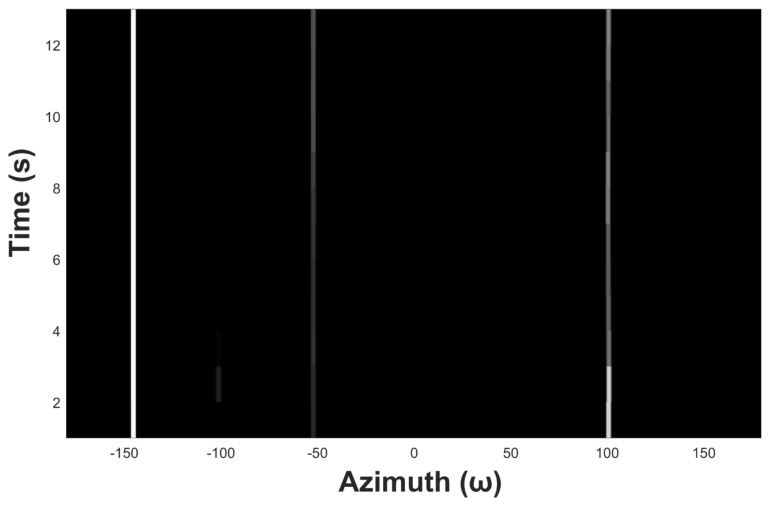
An example of azimuth recording diagram.

**Figure 10:**
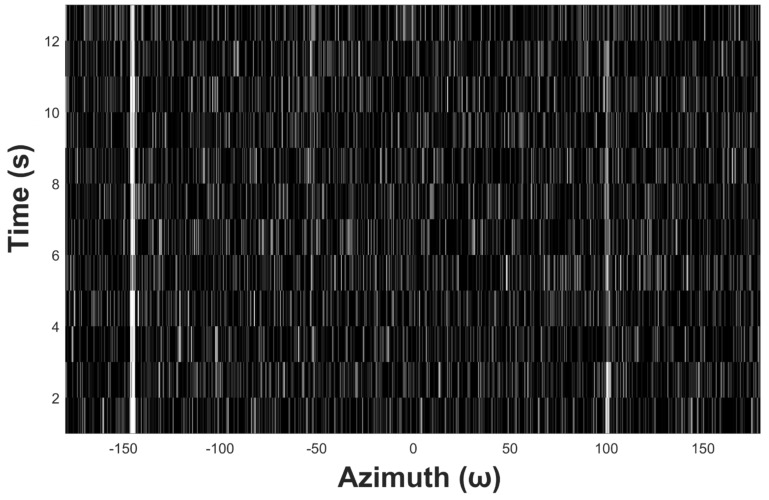
Azimuth recording diagram of underwater acoustic target with noise.

Figure 9 shows an example of azimuth recording diagram of three underwater acoustic targets in the ideal state. The observation time is 13 s, the snap time interval $t_{snap}$ is 1 s, the azimuth angle range is from −180° to 180°, and the azimuth observation interval $\Delta \theta_{step}$ is 0.5°. Figure 10 shows the noised azimuth diagram. It can be seen that the recognizability of the three trajectories becomes much weaker. The weakest trajectory in the middle is completely submerged in the noise and is unrecognizable for the naked eyes.

**Figure 11:**
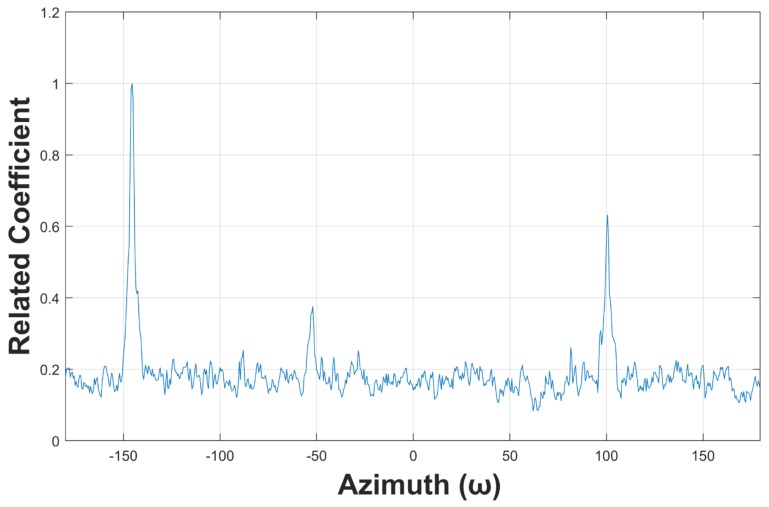
Template matching results.

**Figure 12:**
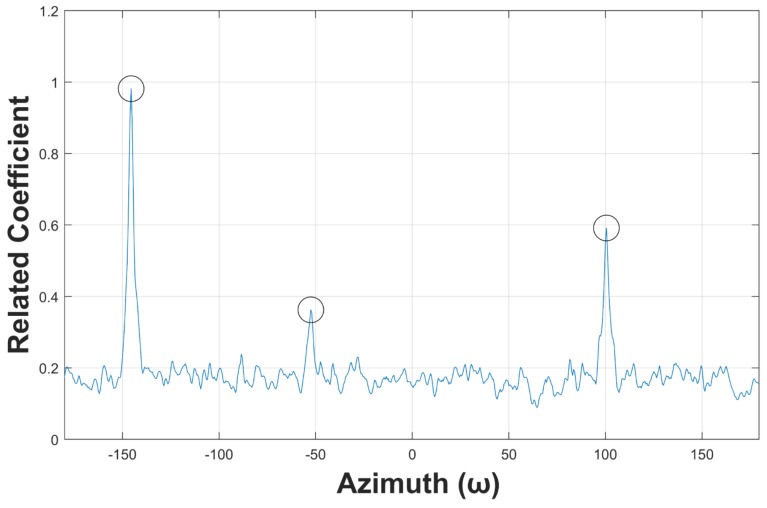
Azimuth estimation results.

Figure 11 plots the matching results of Figure 10, in which three peaks can be easily observed. That is, the ambient noise is effectively mitigated by using the optimal matched filter presented in this paper. Figure 12 displays the smoothed matching results, which is helpful to avoid fault detection by suppressing the interference peaks. The detected targets are marked with black circles. Detecting these peaks is relatively easy, because the trajectory enhancement method presented in this paper enables the threshold ϵ to have a large confidence interval.

### 5.2. Target Tracking

**Figure 13:**
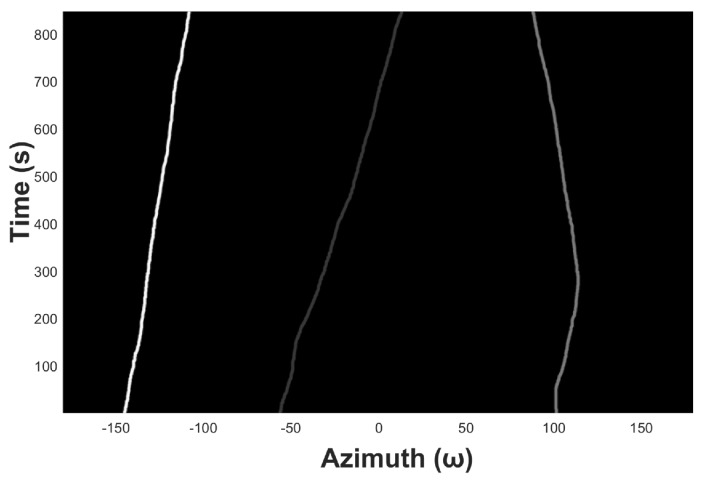
The original azimuth recording diagram.

**Figure 14:**
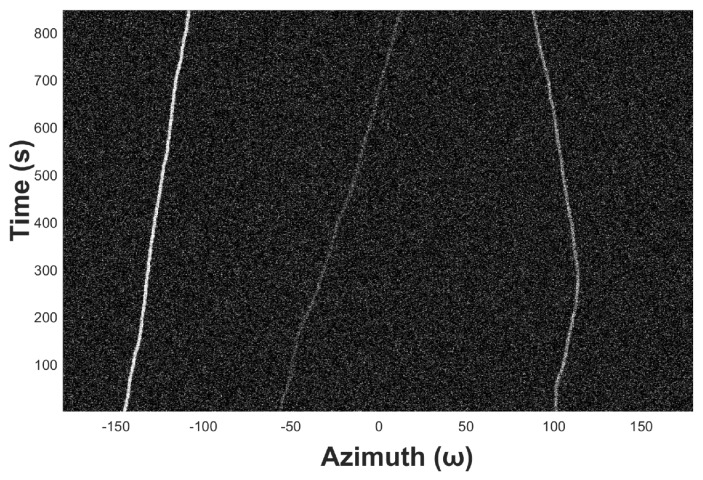
The azimuth recording diagram noised by using Gaussian white noise.

In this subsection, we evaluate the proposed target tracking method with simulated data. Three target azimuth trajectories (see Figure 10) are first detected then tracked. Figure 13 shows the original simulated azimuth recording diagram, whose observation range is from −180° to +180° with a step of 0.5°. It is supposed that three acoustic targets are captured, and their normalized power levels are 1, 0.2, and 0.5, respectively. Figure 14 displays the azimuth recording diagram noised by the white Gaussian noise.

**Figure 15:**
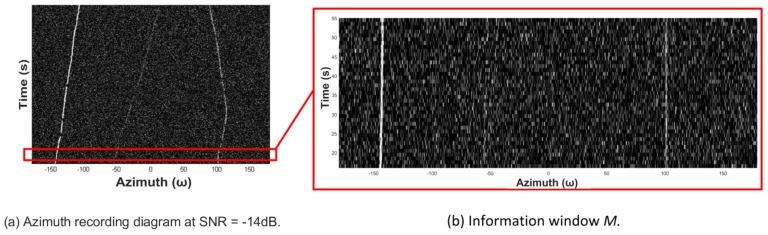
Information window extraction.

The experiment is formed within the environment of SNR = −14 dB, and the time period of the size of ROI *M* is reset as 40 s. Figure 15a displays the diagram to be processed, in which we highlight the ROI *M* with red box and zoom in it in Figure 15b. The trajectory in the middle is too weak to be detected in Figure 15b by the naked eye.

**Figure 16:**
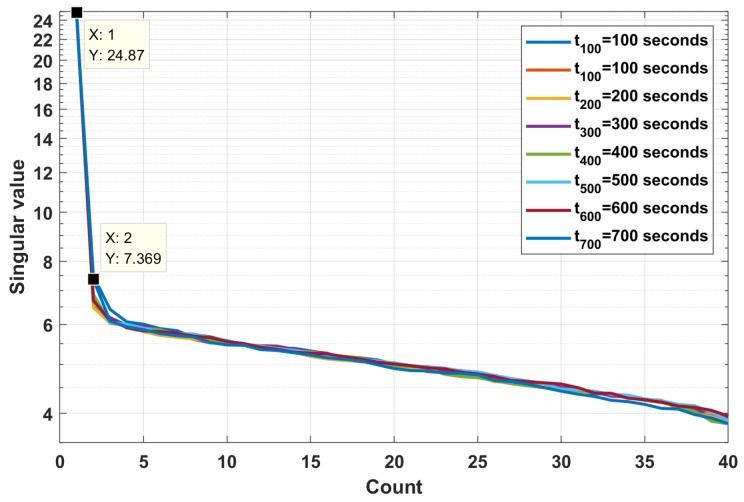
Singular value vectors.

**Figure 17:**
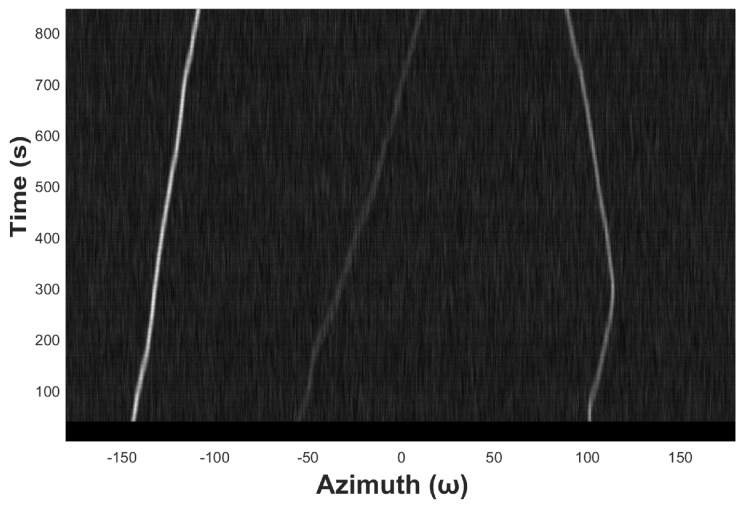
PCA map.

Figure 16 plots the singular values of the PCA spectrum at t=100,200,…,700 s. The first singular values are far above the others, demonstrating that the first subspace of *M* can be considered as the unique principal component, which corresponds to the target subspace. Figure 17 shows the extracted PCA map of the azimuth recording diagram. The image-level noise is well mitigated, and the visual effect of the diagram is significantly improved compared to the noised diagram shown in Figure 14.

**Figure 18:**
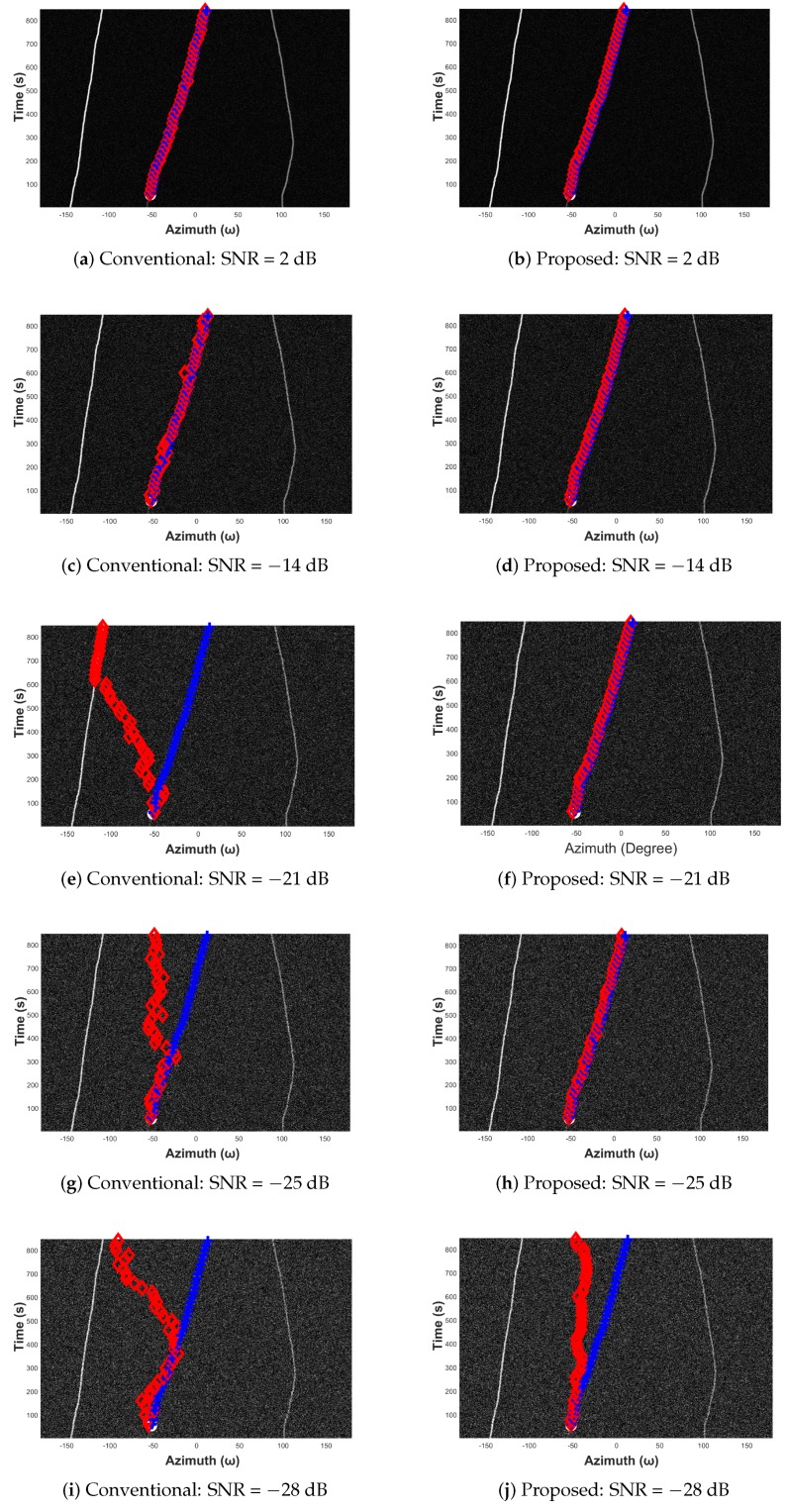
Acoustic tracking results at SNR = 2, −14, −21, −25, −28 dB.

Next, the target tracking stability is evaluated by comparing the tracking results of the conventional and the proposed methods at different noise levels. We simulate the ambient noise with the white Gaussian noise. The variances of the noise are set as 0.01,0.05,0.1,0.15, and 0.2, and their image level signal-to-noise ratios are approximately 2,−14,−21,−25, and −28 dB. Figure 18 displays the comparison results. The white circles are the initial target azimuths of the weakest target, and the real trajectory of the target is marked with blue plus signs and the tracked results the red diamonds. In Figure 18a,b, the noise level is lower than the signals (SNR = 2 dB), and either the original or proposed method is able to track the target successfully.

With the increasing of the noise level, the gap of the stability performance between the original and proposed methods becomes more and more significant. At SNR = −14 dB, we can see that the tracking result of the proposed method shown in Figure 18d is as stable as Figure 18c, whereas in Figure 18c some considerable deviations occur due to the influences of the noise on the original method. In Figure 18e,g,i, the conventional method fails completely but the proposed method still works well, with some minor errors.

**Figure A1:**
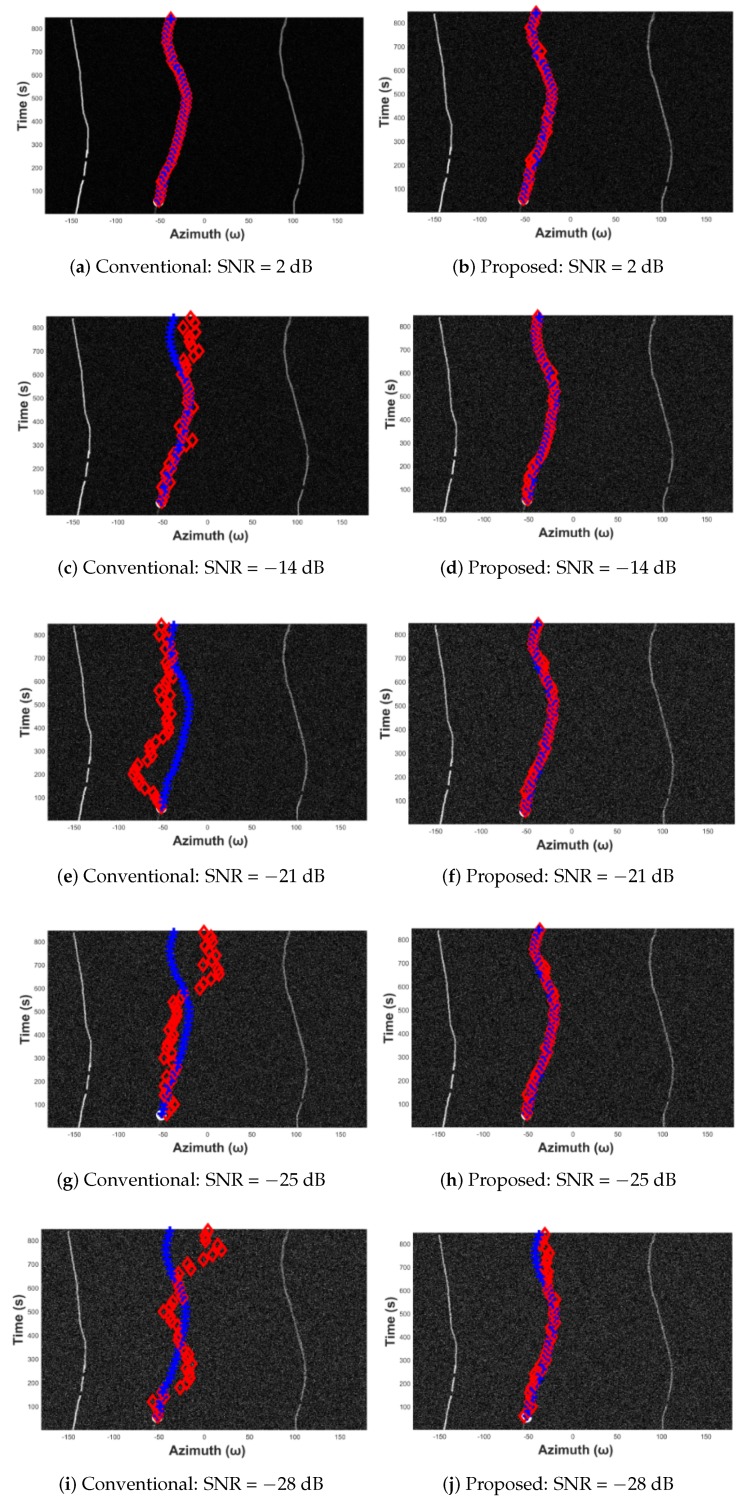
Experiment 1: tracking results at SNR =2,−14,−21,−25,−28 dB.

**Figure A2:**
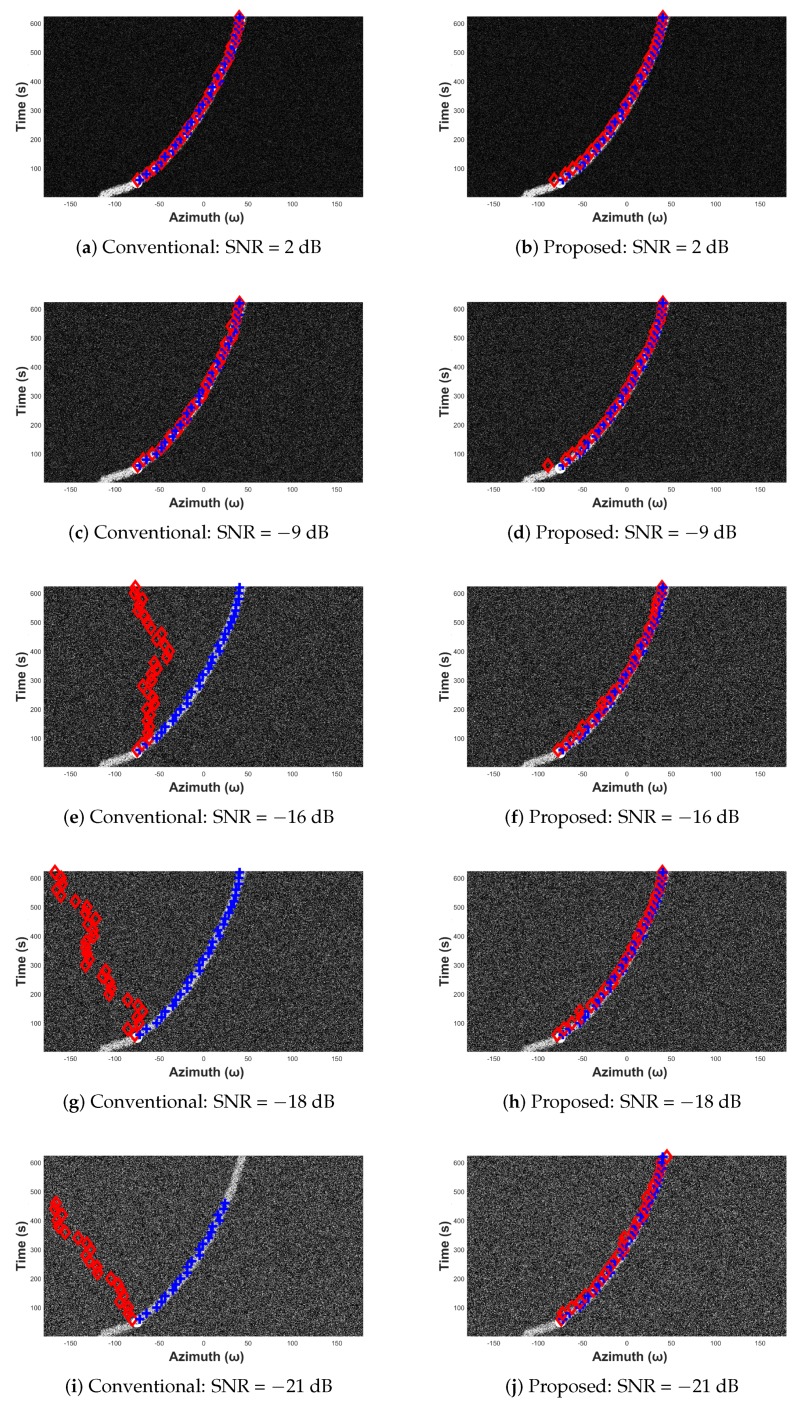
Experiment 2: tracking results at SNR =2,−9,−16,−18,−21 dB.

**Figure A3:**
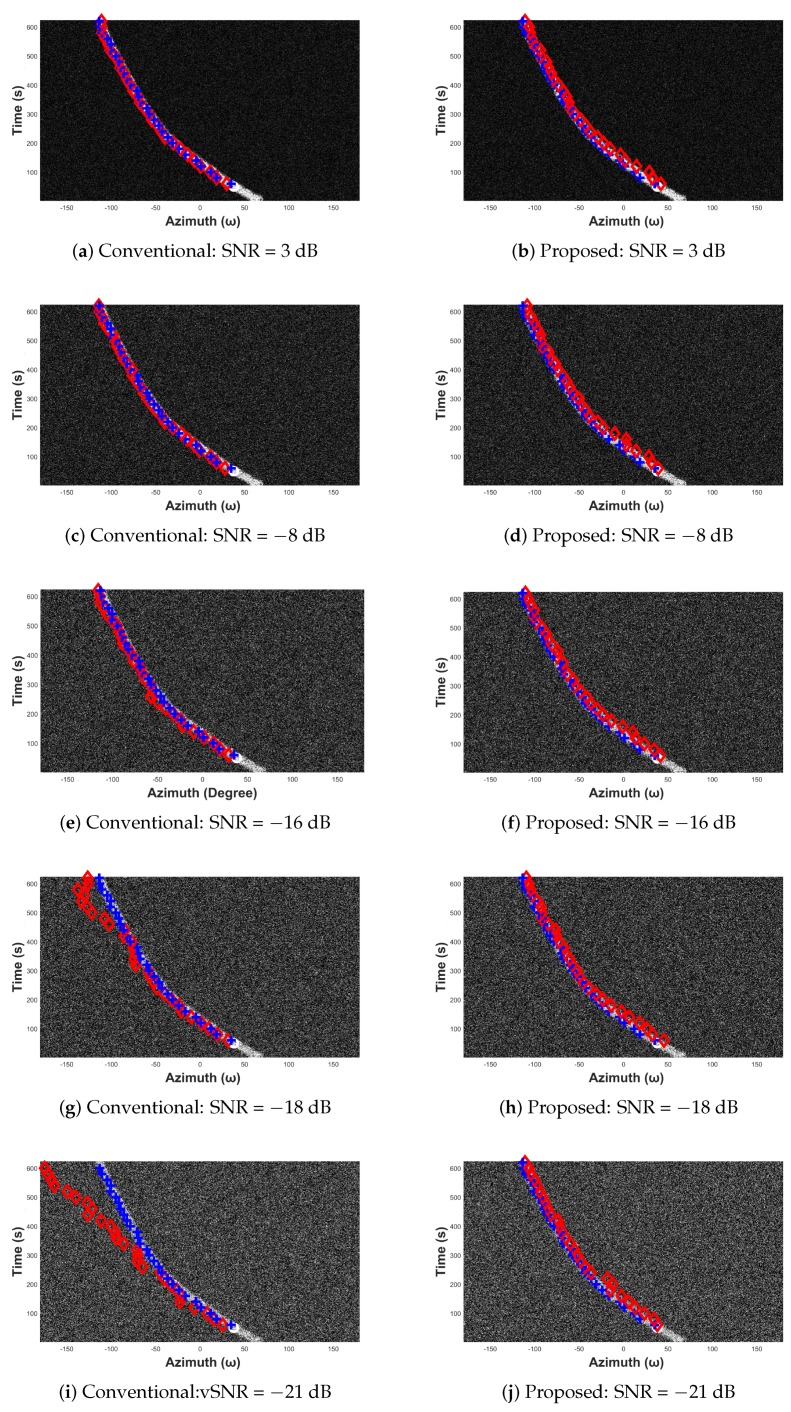
Experiment 3: tracking results at SNR =3,−8,−16,−18,−21 dB.

**Figure A4:**
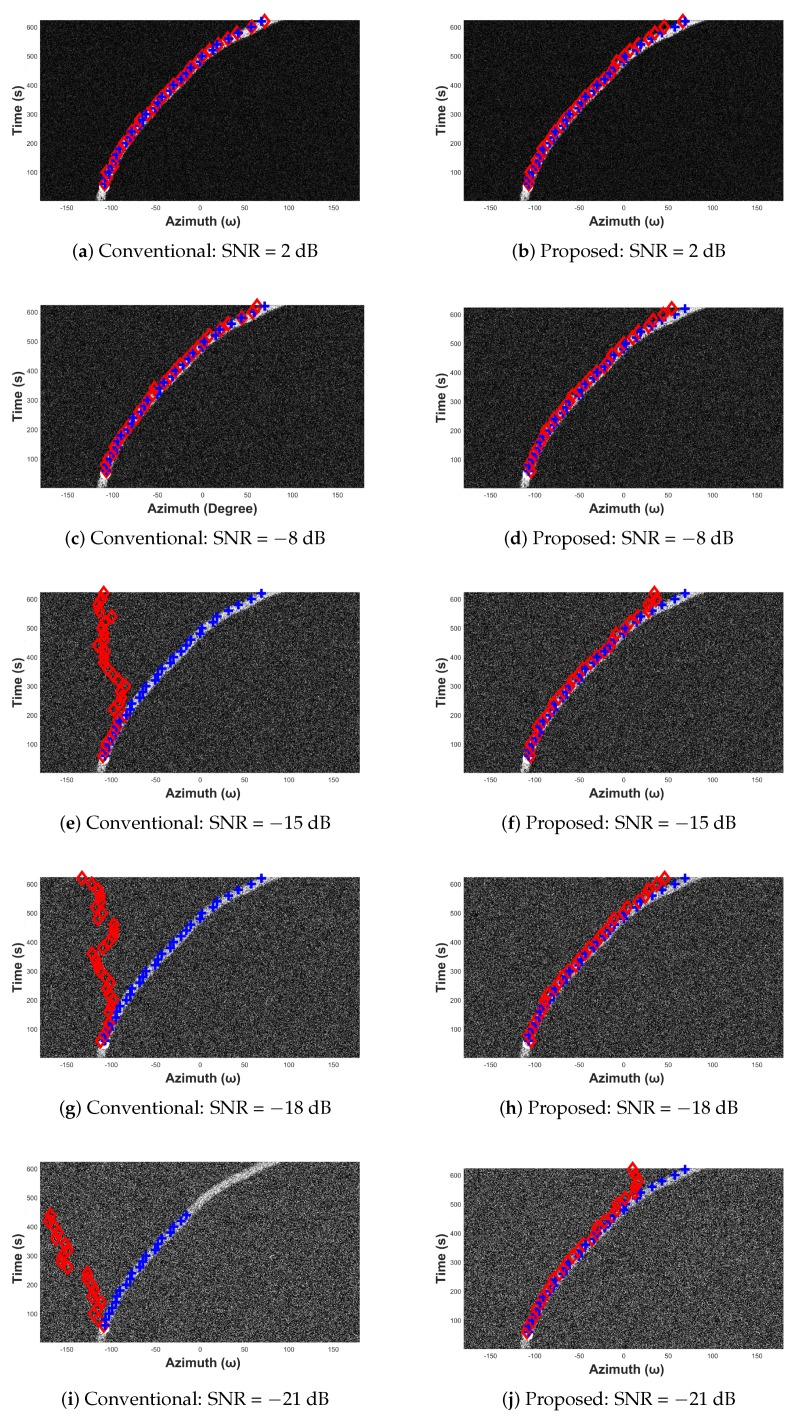
Experiment 4: tracking results at SNR =2,−8,−15,−18,−21 dB.

**Figure A5:**
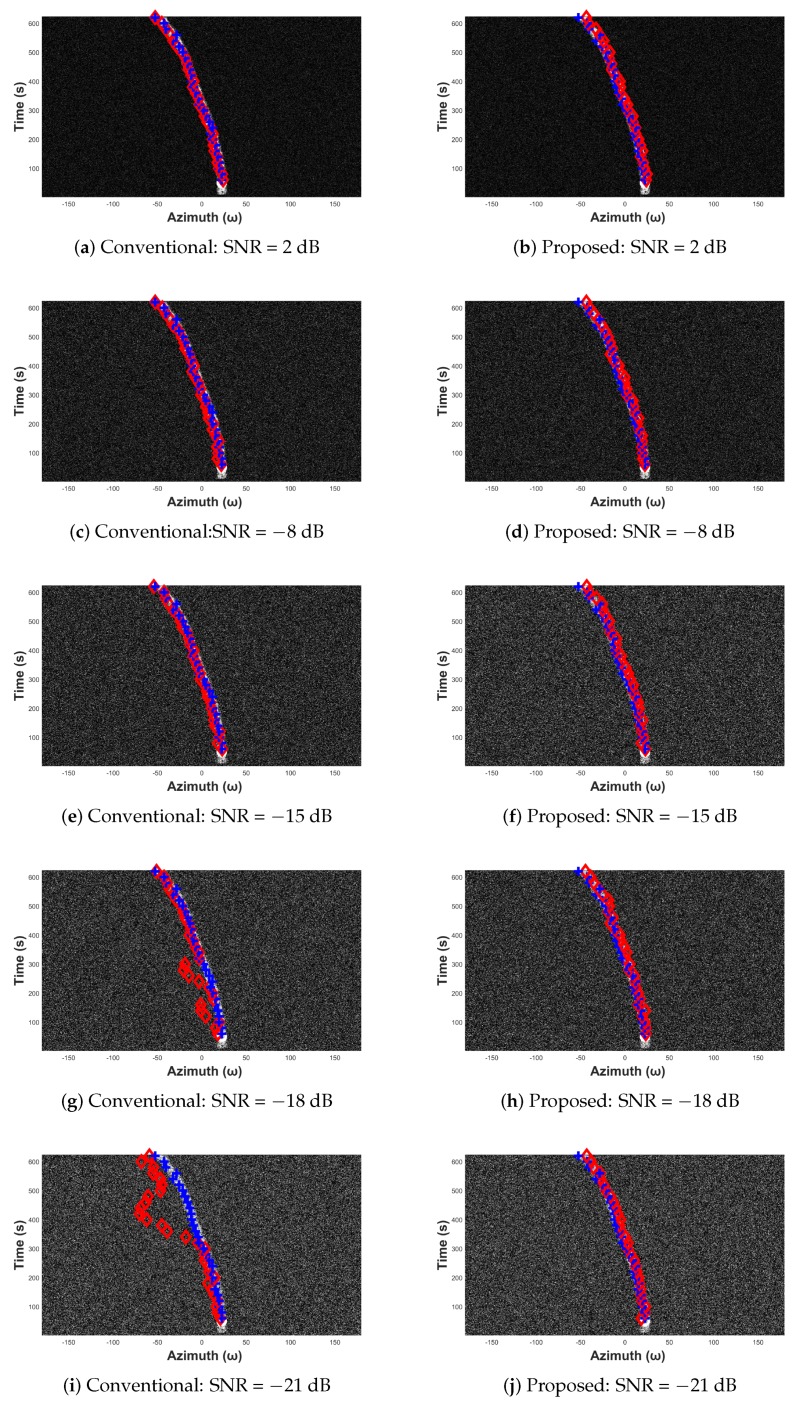
Experiment 5: tracking results at SNR =2,−8,−15,−18,−21 dB.

Finally, the acoustic target is lost in the PCA-enhanced diagram when SNR = −28 dB (see Figure 18j). Overall, the evaluation results of this subsection demonstrate that the original acoustic tracking method is prone to be influenced by the ambient noise on the azimuth recording diagram, such that the azimuth corresponding to a probabilistic noise peak might be mistaken as the azimuth of the target. On the contrary, PCA is able to effectively suppress image noise and achieve stable tracking results, and the performance gap between the two methods is obvious. This conclusion is further verified with five repeated experiments, within which five azimuth recording diagrams having different trajectories are used in order to evaluate the performance of the proposed method with different target movement situations. The experiments are displayed in Figure A1, Figure A2, Figure A3, Figure A4 and Figure A5 in Appendix A.

### 5.3. Accuracy Evaluation

As discussed in Section 5.2, PCA may migrate the azimuth trajectories of the targets, resulting in constant azimuth errors, especially for fast-moving targets. Therefore, an error correction process is performed after the PCA-based tracking to achieve high accuracy. This subsection evaluates the accuracy performance of the proposed method by comparing the tracking results before and after error correcting.

**Figure 19:**
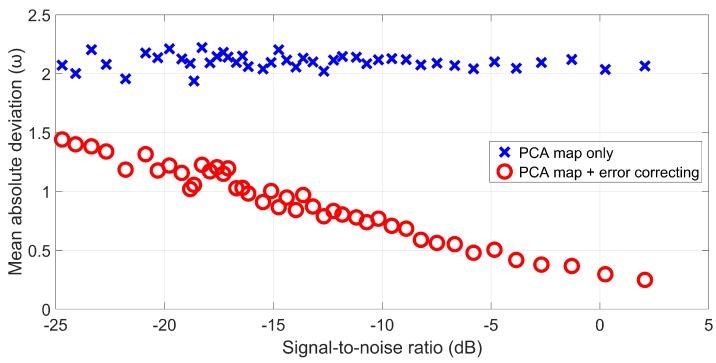
Evaluation results of accuracy performance.

We quantify the accuracy performance by measuring the mean absolute deviations of each running within different SNR environments. In order to obtain an unbiased conclusion, the measuring process is repeated three times and the average results are considered as the azimuth errors at different noise levels. Figure 19 compares the tracking results before and after error correcting. As expected, the azimuth error of the PCA-only curve can be approximated to a horizontal line, demonstrating that PCA migrates the target tracing. On the other hand, after error correcting, the mean absolute deviation decreases with the increasing of SNRs linearly.

Within the SNR measure scope of Figure 19, the error correcting method of this paper leads to lower errors than the PCA-only tracking, and it seems that the accuracy of the former will be higher than the latter if SNR is low enough. However, it should be noted that all the discussions regarding the accuracy performance are based on the hypothesis that the target is tracked successfully and stably. According to our measurements, the PCA-only tracking loses the targets at SNR ≈ −25.5 dB, which is still far away from the noise level threshold where the two methods change places. Consequently, the proposed error correcting method is able to well compensate the weakness of the PCA-based diagram enforcement method regarding the accuracy within its effective SNR range.

### 5.4. Temporal Efficiency

Acoustic target tracking is a typical real-time application, being constrained by the time interval of the data sampling. In this paper, the processing time of each snap must be shorter than the snap interval if the real-time processing capacity is desired. This subsection estimates the running time of the proposed method. We base the algorithm implementing on Matlab 2017b within 64-bit Windows-10 operation system. The processor is Inter (R) Core (TM) i7-8550U, CPU @1.80 GHz. Because the extraction process of PCA spectrum possesses high data dependency, resulting in low parallelism, we do not make any parallel optimizations on the final implementation.

**Figure 20:**
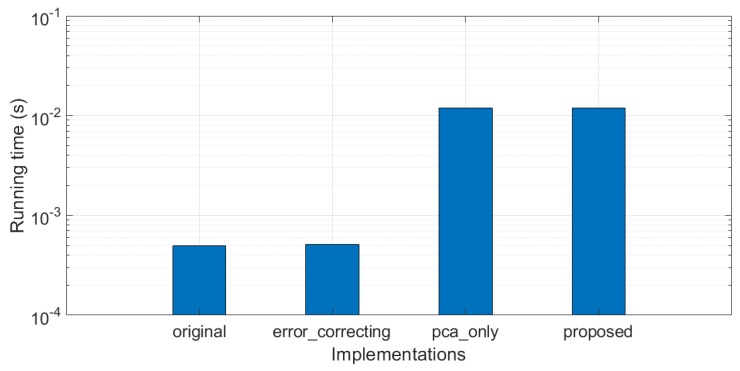
Comparison of computation time among the related methods.

The running time measurement includes the PCA enforcement and tracking processes of each snap. Figure 20 compares the measurement results of four different implementations. “**original**” performs the target tracking directly on the original azimuth recording diagram without any enforcements. “**error_correcting**” optimizes the tracking results of the “**original**” version by using the proposed error correcting method. “**pca_only**” tracks the target in the PCA-enforced diagram without error correcting. Finally, “**proposed**” version combines the PCA enforcement and error correcting.

In Figure 20, it can be first found that the proposed error correcting method does not use too much more computation resource compared to the original. Second, the cycle of PCA is very time-consuming. The running time increases by more than 24 × ($4.95\times 10^{-4}$ vs. $1.2\times 10^{-2}$ s per snap). Consequently, if the real-time processing capacity is desired, the time interval of the snaps should be greater than $1.2\times 10^{-2}$ s. Considering that the time interval of the snaps is usually set as multiple seconds, the hardware resource cost of the proposed method can satisfy the requirements of real-time processing with the up-to-date computation devices.

## 6. Discussion and Conclusions

This paper presents a post-framework for the azimuth recording diagram based acoustic target detection and tracking. The presented approach successfully realizes automatic target detections and highly robust target tracking by incorporating the image processing methods into the passive sonar information processing modalities. First, we base the automatic target detection on the 2-D template matching. A feasible target trajectory template generation method is developed, allowing for specializing the template set for different sensor array designs. Next, inspired by the idea of separating the array signal into the signal and noise subspaces, the target trajectories are significantly enhanced. Based on the separation of useful information subspace obtained by singular value decomposition, the stability of target tracking is greatly improved. Additionally, we find that the azimuth migrations constantly occur if only the PCA spectrum is used. This is because the PCA spectrum transform alters the azimuth tracking constraint from energy optima to that of the dependency of the recorded azimuths. An error-correction strategy is therefore specifically designed by combining the PCA-enhanced and original azimuth recording diagram. The simulation experiments demonstrate that the proposed method can greatly improve the stability of the azimuth tracking technique in underwater acoustic applications.

In the future, improvements will be made regarding the accuracy of azimuth tracking. Additionally, the proposed method will be further evaluated in sea trials. 

## References

[1] Vaccaro R.J. (1998). The past, present, and the future of underwater acoustic signal processing. IEEE Signal Process. Mag..

[2] Akyildiz I.F., Pompili D., Melodia T. (2005). Underwater acoustic sensor networks: Research challenges. Ad Hoc Netw..

[3] Sozer E.M., Stojanovic M., Proakis J.G. (2000). Underwater acoustic networks. IEEE J. Ocean. Eng..

[4] Krim H., Viberg M. (1996). Two decades of array signal processing research: The parametric approach. IEEE Signal Process. Mag..

[5] Muzic R.F., Nelson A.D., Miraldi F. (1993). Temporal alignment of tissue and arterial data and selection of integration start times for the H_2_^15^O autoradiographic CBF model in PET. IEEE Trans. Med. Imaging.

[6] Yang T.C. (2018). Deconvolved Conventional Beamforming for a Horizontal Line Array. IEEE J. Ocean. Eng..

[7] Szalay Z., Nagy L. Target modeling, antenna array design and conventional beamforming algorithms for radar target DOA estimation. Proceedings of the 2015 17th International Conference on Transparent Optical Networks (ICTON).

[8] Burg J.P. Maximum entropy spectral analysis. Proceedings of the 37th meeting of the Annual International Society of Exploration Geophysicists Meeting.

[9] Capon J. (1969). High-resolution frequency-wavenumber spectrum analysis. Proc. IEEE.

[10] Kay S.M., Marple S.L. (1981). Spectrum analysis a modern perspective. Proc. IEEE.

[11] Yang T.C., Yang W.B. Low signal-to-noise-ratio underwater acoustic communications using direct-sequence spread-spectrum signals. Proceedings of the OCEANS 2007—Europe.

[12] Laot C., Coince P. Experimental results on adaptive MMSE turbo equalization in shallow underwater acoustic communication. Proceedings of the OCEANS’10 IEEE SYDNEY.

[13] Cannelli L., Leus G., Dol H., van Walree P. Adaptive turbo equalization for underwater acoustic communication. Proceedings of the 2013 MTS/IEEE OCEANS.

[14] Li H.Y., Yin F., Li C. A High-Accuracy Target Tracking Method and Its Application in Acoustic Engineering. Proceedings of the 2019 IEEE 4th International Conference on Signal and Image Processing (ICSIP 2019).

[15] Berger C.R., Zhou S., Preisig J.C., Willett P. (2010). Sparse channel estimation for multicarrier underwater acoustic communication: From subspace methods to compressed sensing. IEEE Trans. Signal Process..

[16] Cam H., Ucan O.N., Ozduran V. (2011). Multilevel/AES-LDPCC-CPFSK with channel equalization over WSSUS multipath environment. AEU-Int. J. Electron. Commun..

[17] Fuxjaeger A.W., Iltis R.A. (1994). Acquisition of timing and Doppler-shift in a direct-sequence spread-spectrum system. IEEE Trans. Commun..

[18] Lago T., Eriksson P., Asman M. The Symmiktos method: A robust and accurate estimation method for acoustic Doppler current estimation. Proceedings of the OCEANS ’93.

[19] Burdinskiy I.N., Karabanov I.V., Linnik M.A., Mironov A.S. Processing of phase-shift keyed pseudo noise signals of underwater acoustic systems with the Doppler effect. Proceedings of the 2015 International Siberian Conference on Control and Communications (SIBCON).

[20] Bartlett M.S. (1948). Smoothing Periodograms from Time Series with Continuous Spectra. Nature.

[21] Shan L., Dejun W., Haibin W. (2016). An approach to lofargram spectrum line detection based on spectrum line feature function. Tech. Acoust..

[22] Zhang H., Li C., Wang H., Wang J., Yang F. Frequency line extraction on low SNR lofargram using principal component analysis. Proceedings of the 2018 IEEE 14th International Conference on Signal Processing (ICSP 2018).

[23] Zhen L., Li W., Zhao X. (2018). Feature Frequency Extraction Based on Principal Component Analysis and Its Application in Axis Orbit. Shock Vib..

[24] Wang M. (2014). An Improved Image Segmentation Algorithm Based on Principal Component Analysis. Lect. Notes Electr. Eng..

[25] López-Rodrguez P., Escot-Bocanegra D., FernándezRecio R., Bravo I. (2015). Non-cooperative target recognition by means of singular value decomposition applied to radar high resolution range profiles. Sensors.

[26] Demmel J., Veselic K. (1992). Jacobi’s Method is More Accurate than QR. SIAM J. Matrix Anal. Appl..

[27] Drmac Z. (1999). A posteriori computation of the singular vectors in a preconditioned Jacobi SVD algorithm. IMA J. Numer. Anal..

